# Salivary oxytocin, cognitive anxiety and self-confidence in pre-competition athletes

**DOI:** 10.1038/s41598-021-96392-7

**Published:** 2021-08-19

**Authors:** Irene La Fratta, Sara Franceschelli, Lorenza Speranza, Antonia Patruno, Carlo Michetti, Paolo D’Ercole, Patrizia Ballerini, Alfredo Grilli, Mirko Pesce

**Affiliations:** Medicine and Health Science School, University G. d’Annunzio, Via dei Vestini, 31, 66100 Chieti, Italy

**Keywords:** Predictive markers, Emotion

## Abstract

It is well known that soccer sport has the potential for high levels of stress and anxiety and that these are linked to Cortisol (C) variations. To date, much research has been devoted to understanding how Oxytocin (OT) can affect anxiety in response to a challenge. The aim of this study was to investigate, in 56 young male soccer players, the psychophysiological stress response 96 and 24 h before one soccer match of a tournament, in order to establish whether athletes who won or lost, show different levels of C and OT or expressions of competitive state anxiety subcomponents. We found that winners had significantly lower Cognitive anxiety and higher Self-confidence scores than losers. Also, significant differences between winners and losers in C and OT concentrations were observed, with higher OT levels in who has won and higher C levels in who has lost. Our results showed interesting associations between OT, C, anxiety feelings, and the outcome of competition.

## Introduction

Soccer is the world's most popular sport^1^. The soccer sport environment is classified within the most competitive, featured by high mental and physical demands for the athletes. In accordance with other sport contexts, the competition is personally meaningful, and it is typically characterized by several emotions and feelings. The outcome is unknown before the start (uncertainty), and effort is required to fulfill athletic potential. The influence of emotions in soccer is essential for performance because athlete experience anxiety and stress when trying to achieve high performance^2^. Athletes' emotional responses to competition have been studied within the specific area of sport psychology, with numerous researches focused on competitive state anxiety. It is fundamental for the athlete manage this emotion to improve the adaptability to competition^3^.

The “Multidimensional Theory of Competitive Anxiety” classified competitive anxiety into Cognitive and Somatic anxiety, and Self-confidence. In this framework, Cognitive anxiety (CA) is caused by negative self-evaluation and/or negative expectations toward victory, representing the mental component of anxiety. The experience of Somatic anxiety (SA) develops directly from arousal and explains the physiological and affective elements of anxiety. In turn, the level of perceived readiness to compete and confidence is associated to Self-confidence (Self-C)^4^. In agreement with this, Filaire et al. (2009), investigating the physiological and psychological states of tennis players in the first match day during a tournament, found that loser athletes were characterized by higher CA and Self-C scores than winners^5^. The losers, also revealed higher SA scores. Similarly, Strahler et al. (2010), described in competitive ballroom dancers, a significant increase in CA and SA scores before an official competition^6^.

The expression of different emotions in the athletic environment, is influenced by several central and peripheral biological factors, that involved the activation of neurological, immune and endocrine mechanisms^7, 8^. Hormonal variations and psychological states could be useful to monitor the stress response in relation to soccer match performance^9^. Generally, salivary cortisol (C) is frequently used as a biomarker of psychological stress. C plays a central role in the physiological and behavioral response to a physical challenge or to a psychological stressor, that induces the activation of the Hypothalamic Pituitary Adrenal axis (HPA), and hormone release from the adrenal cortex^10^. The level of C may affect the performance, rising closely to an official competition^11^. Higher early-morning anticipatory salivary C levels were associated with winning performance during triathlon competition^12^. On the other hand, higher anticipatory salivary C level characterized kickboxing athletes who lost, suggesting that excessive elevations in C, lead to poor performance, interfering with some cognitive processes^8^.

Oxytocin (OT) is a mammalian neuropeptide produced by the magnocellular and parvocellular neurons of the paraventricular nucleus and the supraoptic nuclei of the hypothalamus^13^. OT acts on many peripheral organs, determining among others, gastrointestinal, reproductive, and cardiovascular effects^14^. In recent times, it has been highlighted that its levels appear to be related to modulation of emotional expression in healthy individuals, influencing emotional and social behavior^15, 16^. Several studies highlight, indeed, how OT can play an important role in simplifying prosocial behavior in humans^17, 18^. One way OT promotes affiliative behavior, is through actions on the HPA axis. Studies carried out on humans, support the idea that OT attenuates the activity of the HPA axis, resulting in lower levels of C under acute stress^19–22^. However, in sport context, few studies on animals and humans have analyzed OT response to exercise^23, 24^, and OT response to soccer competitions has never been investigated.

In contrast to the collection of blood samples, obtaining saliva samples is less-invasive, especially in sport contexts. The assessment of salivary composition can provide a feasible and reliable tool to monitor hormones, and could provide a plasma-comparable method^25, 26^. Measuring C in saliva can successfully offer a reference for blood C levels. Significant correlations have been indeed reported between blood and salivary C concentrations at rest^27^. Nevertheless, saliva OT samples have been reliably measured in several studies^28, 29^, indicating that salivary OT is a good biomarker with which to monitor central OT function^30^.

Taking into account the above considerations, we aimed to investigate hormonal responsiveness and competitive state anxiety in relation to an official competition in winners and losers. We hypothesize a possible role of OT and C in the modulation of stress response before the soccer competition. Considering C as the “stress hormone”, the purpose was to investigate a relationship between state anxiety and the anticipatory C level relating to performance outcome. In addition, we speculate a role of OT in mediating stress response, due to its negative association with state anxiety under the same condition. We candidate these hormonal and psychological variables as potential predictors of victory or defeat.

Psychological assessment was carried out employing psychometric tests to determine levels of competitive anxiety, while biological variables were measured by taking saliva samples.

## Materials and methods

### Ethics statement

The study received institutional ethical approval from the Ethics Committee of G. d’Annunzio University, Chieti, Italy (Prot. n. 1764), and was in accordance with the Declaration of Helsinki. The study adheres to the APA Ethical Standards for the treatment of human participants.

### Participants

Participants included fifty-six young male soccer players of the Youth Sector players of River '65 Elite Soccer School (Chieti, IT). The average age of players was 17.57, SD = 0.49. Prior to the experiment, all participants provided written informed consent from parents to take part in the study. On average the players trained for 8 h per week, which ended with one league game on a weekend. Training content typically involved endurance, strength training and technical/tactical preparation sessions. Volunteers were invited to a preliminary screening session based on a full medical history and examination, the assessment of dietary habits as well as tobacco and alcohol consumption. The participants of the study reported no chronic or acute illness (including periodontal disease), and were in good health in the beginning. All subjects were asked to stay away from any meals and beverages at least 1 h prior to testing time. The average Body Mass Index (BMI) based was within the normal range (M = 22.16, SD = 1.47).

### Study design

**Figure 1:**
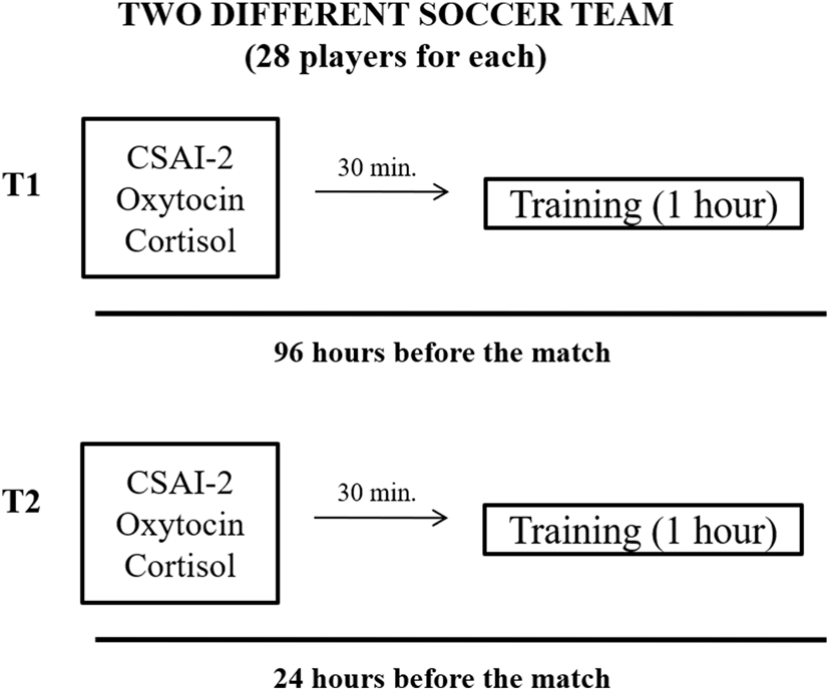
Flow chart of the experimental design.

The study included two different soccer youth team (28 players for team), who participated in a tournament during the normal competitive season. The agonistic season was prefer to 2018–2019, prior to the beginning of the Covid-19 pandemic. The assessment was carried out during a training week, before a first match of a soccer tournament. Players were given psychological tests and salivary samples. Psychometric tests and the salivary samples was carried out simultaneously 30 min before the beginning of the training session, according to the following schedule (Fig. 1):

- 96 h before official soccer match;
- 24 h before the official soccer match.

Considering the circadian drift of endocrine indicators, saliva samples were collected between 2:00 and 5:00 p.m.

### Saliva sampling

Saliva samples were collected using the Salivette system (Sarstedt Co., Nümbrecht, Germany), 30 min before the training session. Each of the samples was collected by having the participant place a cotton swab under the tongue for 4 min.

To avoid contamination of saliva with blood, participants were instructed not to brush their teeth before the saliva sampling. A part from these restrictions, participants were free to follow their normal daily routine on the sampling days. Saliva samples were stored at − 80 °C until biochemical analysis. In order to determine blood contamination of saliva samples, a Salivary Blood Contamination Enzyme Immunoassay Kit (Salimetrics, State College, PA, USA), which measures the level of transferrin in saliva samples was used. According to the findings by Schwartz and Granger (2004), the participants with salivary levels of transferrin ≥ 5 mg/L were excluded. None of them have been excluded^31^.

### Enzyme linked immunosorbent assay (ELISA)

The salivary OT levels were measured using the commercial kit Oxytocin EIA kit (Genprice Inc., San Jose, CA, USA) (Oxytocin ELISA kit, cat. No. K048). Salivary C levels were measured using the commercial kit ELISA (Thermo Fisher Scientific, Waltham, MA, USA) (Cortisol ELISA kit, cat. No. EIAHCOR). The measures were performed according to the instructions of the producer. Plates were scanned using a specialized Charge Coupled Device cooled tool. The integrated density values of the spots of known standards were used to generate a standard curve. Density values for unknown samples were determined using the standard curve for each analyte to calculate the real values in pg/mL or ng/mL. All steps were performed twice and at room temperature. The OT assay sensitivity was equal to 1.7 pg/mL, for the C it was equal to 17.3 ng/mL. The intra- and inter-assay reproducibility was > 90%. Duplicate values that differed from the mean more than 10% were considered suspect and therefore repeated. We did not reported missing values in salivary evaluation of OT and C.

### Psychological assessment

The measurement of anxiety was performed by applying the Italian version of Competitive State Anxiety Inventory-2 (CSAI-2)^32^, constructed by Martens, Vealey, Burton, Bump and Smith (1990)^33^. The CSAI-2 is a sport-specific self-report questionnaire comprising 27 items pertaining to three subscales of CA (the fear of negative social evaluation, fear of failure and loss of self-esteem), SA (perception of personal physiological reactions in competitive situations) and Self-C (belief or degree of certainty individuals possess about their ability to be successful in sport). Responses to each item are rated on a scale of 1 (not at all) to 4 (very much so), giving each subscale a range from 9 to 36. High scores on CA, SA and Self-C indicate high levels of anxiety and self-confidence, respectively.

The CSAI-2 inventory shows homogeneity, reliability and sensitivity in measuring psychological characteristics in athletes and has good internal consistency (Cronbach’s alpha coefficients of 0.79–0.90). It is a reliable and valid measure of state anxiety and self confidence^4, 33^.

The internal consistency values (Cronbach’s Alpha) for this research were as follows: 96 h—Winners: CA = 0.79; SA = 0.77; Self-C = 0.95. 96 h—Losers: CA = 0.85; SA = 0.64; Self-C = 0.84. 24 h—Winners: CA = 0.86; SA = 0.67; Self-C = 0.87. 24 h—Losers: CA = 0.89; SA = 0.72; Self-C = 0.85.

### Statistical analysis

The normal distribution of data was tested by the Skewness and Kurtosis measurement, and analyzed respectively with parametric or non-parametric test. The results were reported separately for each time point: 96 and 24 h. The state of CA, SA and Self-C as well as hormones levels, in each experimental time, were analyzed by two way ANOVA for repeated measures followed by the Bonferroni post-hoc test.

In accordance with distribution and the scale of the variables, the co-efficient of Pearson correlation (*r*) was applied to assess the correlations between the endocrine mediators (Δvalue of C and Δvalue of OT), and Δvalue of subcomponents of CSAI-2 scores (ΔCA, ΔSA and ΔSelf-C) (Δemotional state score = emotional state score at 96 h *minus* emotional state score at 24 h) were also evaluated. The ΔCA and ΔSelf-C scores were used as a dependent variable in linear regression analyses, whereas ΔC and ΔOT were respectively used as independent ones.

Statistical analyses were performed using the SPSS 20.0 statistic (SPSS Inc., Chicago, IL, USA) for Windows (IBM). Results are described as means ± SD for each assessment performed in triplicate. All statistical tests were two-tailed and were evacuated at an alpha level of 0.05.

## Results

### Psychological response to competition and match outcome

**Figure 2:**
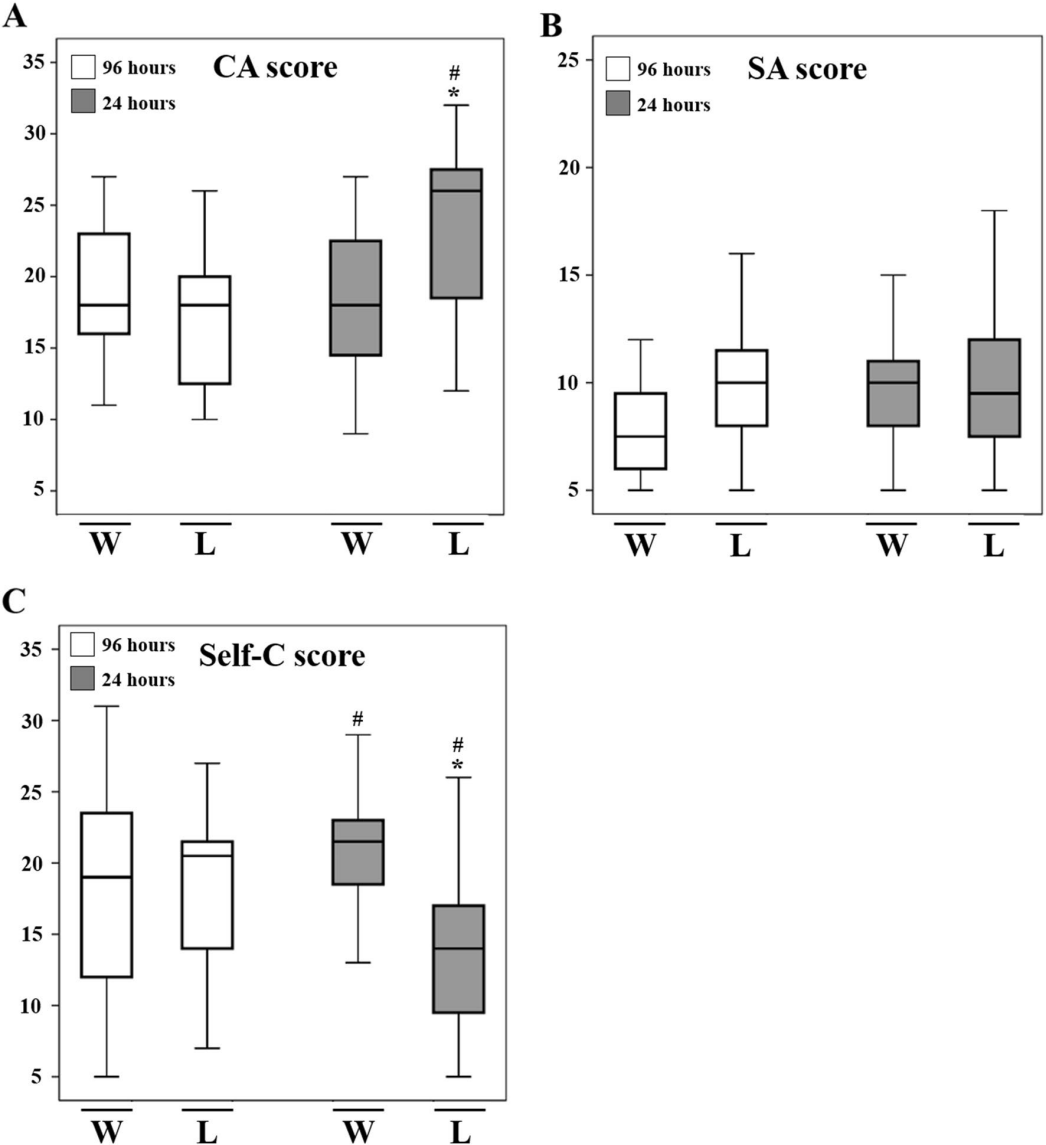
Psychometric evaluation of competitive anxiety. Psychometric evaluation was performed for Cognitive Anxiety (CA), Somatic Anxiety (SA) and Self Confidence (Self-C) in those who won (N = 28), and those who lost (N = 28) at 96 h and 24 h pre-official competition. The CA, SA and Self-C scores were assessed by CSAI-2. Those who lost showed higher CA scores at 24 h pre-official match than those who won. At the same time, losers athletes showed significantly lower Self-C score than winner at 24 h pre-official match. Data are expressed as means ± SD. Significance was obtained with repeated measures ANOVA (1 between the winner-loser factors; 1 within time factor) followed by the Bonferroni post hoc test. ^#^*p* < 0.05 versus previous competition time point; *p < 0.05 versus winner athletes at the same time point.

In 56 male soccer players the intensity of anxiety was assessed by CSAI-2, 96 h and 24 h before the first match of soccer tournament. Psychological variables are presented in Fig. 2.

The repeated measures ANOVA analyses for CA, showed that the effect within subjects was significant. The CA scores increased from 96 to 24 h in all athletes (F_(1, 54)_ = 25.90, *p* < 0.0001). In particular, when we considered the outcome as within factor, CA scores were significantly higher 24 h pre-official competition in athletes who lost compared to relative counterpart at 96 h (24 h vs 96 h, 23.21 ± 5.80 vs 17.04 ± 4.90) and the winning team (23.21 ± 5.80 vs 18.4 ± 4.50) (F_1, 54_ = 35.00, *p* < 0.001) (Fig. 2A). We did not observe any over time variation in SA scores between who won and who lost (F_(1, 54)_ = 1.51, *p* = 0.224, η_p_2 = 0.027) (Fig. 2B). Self-C scores were significantly higher 24 h before the match in athletes who won compared to relative counterpart at 96 h (24 h vs 96 h, 24.86 ± 5.5 vs 21.93 ± 8.11). Conversely, Self-C scores were significantly lower at 24 h in athletes who lost when compared to themself at 96 h before the match (24 h vs 96 h, 16.86 ± 5.44 vs 24.86 ± 5.5) (F_(1, 54)_ = 16.39, *p* < 0.001, η_p_2 = 0.233) (Fig. 2C).

### Hormonal response

**Figure 3:**
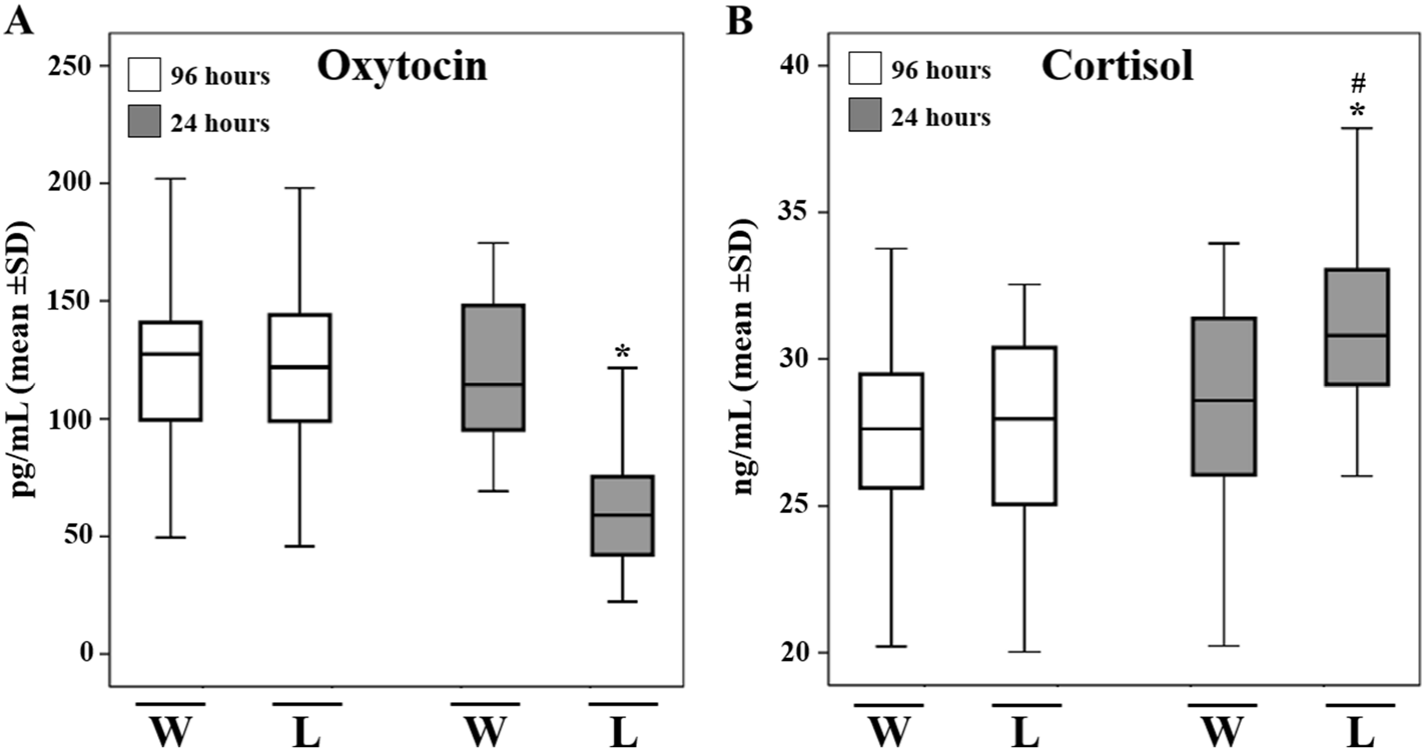
Hormonal response to coming competition. The ELISA measurement of Oxytocin (OT) and Cortisol (C) in winner team (N = 28) and loser team (N = 28) 96 h and 24 h pre-official soccer competition in salivary samples. Those who lost, showed lower OT salivary level 24 h pre-official match than winners (**A**). Athletes who won, showed a significantly higher C salivary levels 24 h pre-official match than losers (**B**). Data are expressed as means ± SD. ^#^*p* < 0.05 versus previous competition time point; *p < 0.05 versus winner athletes at the same time point.

The mean hormonal concentrations are presented in Fig. 3 for winner and loser teams. Soccer players who lost, were characterized by significantly lower salivary level of OT 24 h pre-official soccer competition respect to 96 h (63.3 ± 26.9 pg/mL vs 124.23 ± 41.87 pg/mL) (F_(1,54)_ = 43.46, *p* < 0.001, η_p_2 = 0.446), and the winning team at 24 h (63.3 ± 26.9 pg/mL vs 115.0 ± 36.1 pg/mL) (F_(1,54)_ = 20.70, *p* < 0.001, η_p_2 = 0.277) (Fig. 3A).

On the other hand, winning athletes showed significantly lower levels of C 24 h before the official competition when compared to losing athletes at the same time (28.34 ± 3.7 ng/mL vs 30.89 ± 3.1 ng/mL) (F_(1,54)_ = 5.98, *p* < 0.001, η_p_2 = 0.100). Additionally, the team that won showed no significant variation over time (27.6 ± 3.32 ng/mL vs 28.34 ± 3.7 ng/mL), while a significantly increase in salivary C levels was recorded in athletes who lost compared to 96 h earlier (30.89 ± 3.1 ng/mL vs 27.49 ± 3.4 ng/mL) (F_(1,54)_ = 14.45, *p* < 0.001, η_p_2 = 0.211) (Fig. 3B).

### Associations among measures

We analyzed the possible correlations that occur between the biological variables and the emotional state scores (ΔCA, ΔSA and ΔSelf-C: Δvariable = variable_96 h_ *minus* variable_24 h_) across the time points considered.

Table 1 shows the bivariate correlations between salivary hormones level variation. The analysis showed that age was not related to any variables considered. No significant correlation was found between the ΔSA score and salivary level of hormones as well as the emotional variables at each time point. Significant negative correlation was found between the ΔOT and ΔC salivary levels (*r* =  − 0.570, *p* < 0.01).

**Table 1:** Bivariate correlation among variables.

	Age	ΔOT	ΔC	ΔCA	ΔSA	ΔSelf-C
Age	**–**	− 0.069	− 0.161	− 0.030	− 0.071	− 0.125
ΔOT		–	− 0.570**	− 0.601**	0.130	0.538**
ΔC			–	0.556**	− 0.001	− 0.537**
ΔCA				–	− 0.178	− 0.588**
ΔSA					**–**	− 0.001

Pearson (*r*) correlation between Age, ΔCognitive Anxiety score (ΔCA), ΔSomatic Anxiety score (ΔSA), ΔSelf Confidence score (ΔSelf-C) and salivary hormonal markers in healthy soccer male athletes. The psychometric assessment and biological evaluations were performed at 96 and 24 h pre-official match (N = 56). OT = Oxytocin; C = Cortisol.

Δvariable = variable_96 h_ *minus* variable_24 h_; *p < 0.05; **p < 0.01.

As regards to the salivary levels of the analyzed hormones and their relationship with emotional states, the assessment showed that ΔCA score was positively correlated with ΔC salivary levels (*r* = 0.556, *p* < 0.01), and negatively to ΔOT salivary levels (*r* =  − 0.601, *p* < 0.01). Also, ΔCA score was negatively correlated with ΔSelf-C score (*r* =  − 0.588, *p* < 0.01). On the contrary, the ΔSelf-C score correlated positively to ΔOT salivary level (*r* = 0.538, *p* < 0.01), and negatively with ΔC salivary level (*r* =  − 0.537, *p* < 0.01).

Linear regression analysis was used to determine whether the temporal variation across experimental time points considered of the CA state and Self-C score can be explained by the temporal variation of OT and C levels in saliva ascertained at the same time.

The results are shown in Table 2. ΔCA score, showing significant zero-order correlations with the biological variables, was regressed on ΔOT and ΔC salivary levels in a simultaneous regression analysis, using Age as control variable. The overall model was significant (F_(3,52)_ = 13.00, ANOVA *p* < 0.01), accounting for the 40% of the ΔCA score variance.

**Table 2:** Regression analysis.

Dependent variable	Independent variable	R^2^	R^2^ adjusted	F_(3,52)_	*p*-value
ΔCA	Age, ΔOT, ΔC	0.429	0.396	13.009	0.001
ΔSelf-C	Age, ΔOT, ΔC	0.394	0.359	11.262	0.001

Summary for biological variables predicting ΔCognitive Anxiety (ΔCA) score and ΔSelf Confidence (ΔSelf-C) score. ΔCA score was regressed on Age, ΔOT and ΔC (N = 56; F_(3, 52)_ = 13.00; *p* < 0.001), and ΔSelf-C score was regressed on Age, ΔOT and ΔC (N = 56; F_(3, 52)_ = 11.26; *p* < 0.001).

The ΔSelf-C score was regressed on ΔOT, ΔC using Age as control variable. The model was significant (F_(3,52)_ = 11.26, ANOVA *p* < 0.01), accounting for the 36% of the ΔSelf-C score variance.

## Discussion

In the present study, we sampled players of two different teams at the beginning of the training week (96 h pre) and the day before (24 h pre) an official match of a tournament. The aim of the study was to determine if the variations in psychological state and concentration of the endocrine variables examined, might be predictive of higher performance. To this end, we (1) analyzed the athletes' psychophysiological responses in the pre-competition period, and (2) investigated the relationships between the competitive state anxiety feelings and salivary levels of C and OT in male soccer players.

We here showed that the two soccer team displayed different hormonal and competitive state anxiety responses before an official match. There were changes in CA and Self-C scores, as well as in OT levels, with a drop before the match which ended in defeat and a rise before the match ending in victory. Moreover, we described changes in C levels, with a significant rise before the match ending in defeat.

To assess the competitive anxiety state, we used the CSAI-2 inventory characterized by items focused to have an experiential measure of the anticipatory stress response to competition. This test evaluates the perceived arousal and worries about the outcome.

Winners players' team showed significantly lower levels of CA than losers the day before the match. Nowadays, the association between performance and anxiety is not well clarified, and is somewhat controversial^5, 34^. The level of competitive anxiety may be perceived by some athletes as facilitative and by others as debilitative, showing that its necessary level to attain good performance is subjective^35, 36^. We also described that SA scores did not differ significantly between winners and losers. This supports the proposal that SA is basically reflexive and undergoes a more rapid adaptation as competition approaches. SA scores did not correlated with CA indicating that in our sample CA and SA do not vary at the same time. This is a somewhat unexpected result and it might be explained by considering that SA aims to shows a sudden increase peaking at the start of the competition. We did no assess the state anxiety close to match (e.g. 1 h before) and this could be a limitation of the study; however, although SA is a useful indirect measure of the physiological indices of anxiety, it is of limited theoretical value in explaining the relationship between physiological arousal and performance^4^.

On the other hand, Self-C and CA have more influence on performance because they are self-evaluative and strictly change when performance expectations change. Regarding Self-C scores, winners showed higher score than losers the day before the match, highlighting how greater Self-C can help to better cope with stressful events by modulating feelings of anxiety^37^. Moreover, Self-C negatively correlated with CA scores, suggesting that Self-C influence anxiety interpretation by offering individuals protection against the debilitating effects of stressful situations^38, 39^.

The hormones levels were quantified by saliva samples. In sport, saliva collection can provide a feasible and reliable tool to monitor hormones levels in a short time. Taking a saliva sample, eliminating likely (psycho-social) stress sources, is a non-invasive method which does not interfere with hormonal system activity. While the saliva is considered a validate tool in which measure C^40, 41^, the measure of salivary OT is new-found. To this end, Jong et al. (2015), suggested that in response to stimuli that are known to stimulate peripheral and central release of OT, significant differences in the levels of the neuro-peptide can be detected in saliva^24^.

The losing players experienced an increase in C levels over time, whereas in winning team no significant difference in C levels was found. Regarding sport context, the majority of studies on hormonal response to competition have detected an anticipatory rise in C^42, 43^, showing that circulating C helps to prepare the body for action as part of the physiological stress response. In this way, circulating C, through its influence on cognitive processes, could affect performance and indirectly influence the outcome^44^. Our data support the findings of Cintineo et al. (2019) who found that losers wrestlers were characterized by pre-competition higher levels of salivary C and state anxiety, compared to winners^45^. However, other studies found opposite trend, with higher C levels and CA in winners rather than losers, before competition^46, 47^.

These discrepancies may be related to sport specific factors or individual differences. In this regard, Van Paridon et al. (2017) in a recent meta-analysis showed that generally in all sports, high level competitors exhibit changes in C or little to no reactivity during anticipation of competition^48^. Moreover, another reason may be explained by different coping style, which refer to individual differences related to perceived control over one's environment^49^. In addition, in our study, C concentration and Self-C were significantly and negatively correlated, suggesting that based on the level of C, athletes may experience a reduction in their Self-C, which thus negatively affects their performance^50^.

Drawing first conclusions, we showed that higher anticipatory C response is associated with higher levels of CA, low Self-C and poorer match outcomes. In general, research indicates that moderate elevations in C and anxiety, could be depending on arousal demands to anticipatory energy resources mobilization at the nearing of the competition time. It could reflect also preparatory stress responses triggered when arriving at the competition venue, in detail as responses to a social environment replete with classically conditioned stimuli^51^. Therefore, extreme elevation in C concentrations leads to poor performance plausibly because it interferes with some cognitive processes^52^. Based on the findings of the current study, it can be hypothesized that the higher levels of CA scores, seen in match losers, may have been “too high”, resulting in an overall decrement in performance.

To our knowledge, there are few studies that have evaluated the role of OT in response to a sport challenge. Even though, sporting contest have been considered a reliable field in which study response to stress. Concerning salivary OT, we recorded lower levels in losers at 1 day before the match, comparing to winners at the same time, and respect to themselves at 96 h. Of note, OT concentration was negatively correlated with C levels and CA scores. The association between OT and stress may appear paradoxical. Despite evidence that OT might attenuate stress reactivity and reduce anxiety, there are several findings showing opposite effects. For example, in a recent study, high levels of plasma OT were associated with fewer depressive symptoms and greater maternal sensitivity among postpartum mothers, but only for those women who reported high levels of psychosocial stress^53^. However, it should be emphasized that the hypothalamus and the hippocampus are involved into stress regulation. The hypothalamus projects OTergic neurons to the hippocampus, and the latter possesses high levels of OT receptors. The hippocampus also regulates the secretion of C. Excessive levels of C during chronic stress cause atrophy of the hippocampus, whereas OT has been shown to protect hippocampal neurons from the toxic effects of stress hormone^54^. In fact, lower plasma OT levels were reported to be associated with anxiety symptoms in patients with depression^55^, and symptoms of separation anxiety and depression during pregnancy^56^. Hoge et al. (2012) also reported low plasma OT levels in patients with general social anxiety disorder, during a prosocial laboratory task paradigm^57^.

The relationship between OT and C, it is not well clarified and is somewhat questionable. In this regard, Jong and colleagues (2015), described that salivary OT and C followed a rising parallel trend during a Trier Social Stress Test procedure, depending on oral contraceptive use^24^. On the other hand, there is a strong evidence that OT is associated with lower C level under acute stress^21, 22, 58^. For example, some studies have shown that the increase in OT is linked to relaxation and calm, as well as to the affiliation behavior^55^, through the inhibition of the HPA axis response induced by stress^59^. These results permit us to speculate that the interplay between these hormones is dynamic and sensitive to anticipation of stress or novelty. Furthermore, the anxiolytic and stress-relieving effects of OT may be context-specific and may vary depending on the timing of the perceived stressor^60^.

In the present study, the negative correlation of OT with C and CA, would support that notion of a protective effect of OT under stress. Probably, the dampening effect of OT on C may only occur in tasks that are sufficiently stressful to elicit a strong HPA axis response^20^, such as a sport match. Generally, a challenging situation such as competition, stimulates high response from participants, and the self-interpretation of a stressful stimulus/situation, establishes how the HPA axis responds to that stimulus^61^.

In addition, we found a positive correlation between OT levels and Self-C score. OT-associated Self-C might be within the underpinning of specific social affiliative choices, such as the engagement into potentially risky investments or the alliance with a dominant persons, physically strong^62, 63^. Of note, during stressful events, a positive view of self leads individuals to prefer upward rather than downward social confrontation. As consequence, they are ready to affiliate/compare with individuals potentially perceived as stronger without fear of being “overpowered” by them, at the same time the other is believed as precious resource for self^64^. This phenomena is main relevant in athletic domain, because OT rises in response to perceived social support. This last may be a key factor in facilitating challenge states converging from coaches, team-mates, audiences and significant others^22^.

Before the conclusions, we reported some limitations of the present study: (1) the sample population is small. This limitation has been partly compensated by taking measures over time; (2) the time points chosen (96 and 24 h) are quite long to trigger noticeable preparatory stress responses. In this regard, the choice of the times derives from the final purpose of the study, which is to identify biological markers and emotional states predictors of good performance, helping players to cope with stressful situations, and coaches abling to set up training environments simulating similar psychophysiological responses.

In conclusion, it is reasonable to speculate that OT mitigates stress responses under challenging circumstances, fueling a positive self-view and enhancing social skills in soccer youth players during sport challenge.

## Data Availability

The data that support the findings of this study are available from the corresponding author upon reasonable request.
